# Beyond the Document: A Single-Center Qualitative Study of Survivorship Care Plan Barriers and Opportunities Across Pediatric Oncology Stakeholders

**DOI:** 10.1002/1545-5017.70677

**Published:** 2026-09-10

**Authors:** Molly S. Talman, Emma Fleisher, Megan E. Salwei, Debra Friedman, Laurie Novak

**Affiliations:** 1 Department of Pediatrics, Vanderbilt University Medical Center, Nashville, Tennessee, USA; 2 Department of Biomedical Informatics, Vanderbilt University Medical Center, Nashville, Tennessee, USA

**Keywords:** artificial intelligence, childhood cancer survivors, pediatric oncology, primary care, qualitative research, survivorship care plan

## Abstract

**Background:**

Survivorship care plans (SCPs) summarize cancer treatment and guide risk-based follow-up for cancer survivors, yet remain difficult to create, share, and use. Stakeholder perspectives are needed to inform usable approaches.

**Objective:**

To characterize how SCPs are created, shared, and used in pediatric oncology and identify stakeholder priorities for improving workflows and SCP design.

**Methods:**

We conducted semi-structured interviews with childhood cancer survivors and family members, primary care providers (PCPs), and oncology clinicians at a single academic center. Interviews explored SCP receipt or creation, usefulness, barriers, and communication across care settings. Transcripts were analyzed using inductive thematic analysis, with interpretation informed by a sociotechnical work-system perspective.

**Results:**

Twenty-one participants were interviewed: seven survivor/family participants (one survivor, six caregivers), nine PCPs, and five oncology clinicians. Four higher order themes were identified: (i) Survivorship care remained oncology-centered, reflecting relational continuity developed during treatment and uncertainty about shared-care roles. (ii) SCPs were valued but poorly integrated into clinical workflows. (iii) SCP content reflected standardized documentation more than tailored user needs: families needed understandable information, PCPs needed concise action items and role clarity, and oncology clinicians needed detailed treatment and guideline information. (iv) Stakeholders viewed digital and AI-assisted tools as potentially helpful for reducing manual work and improving access, but emphasized accuracy, transparency, and oversight.

**Conclusion:**

SCP challenges extend beyond the document to surrounding systems of communication, responsibility, and workflow. Future SCPs should be embedded in clinical processes and designed as role-specific, user-centered tools, with technology supporting rather than replacing clinician judgment and relational continuity.

## 1 | Introduction

Survivors of childhood cancer represent a growing population with lifelong health needs related to their prior cancer and treatment. In the United States, approximately 500,000 individuals treated for cancer during childhood are alive today, and most will develop at least one chronic health condition by mid-adulthood [1]. Risk-based survivorship care is therefore essential to identify late effects associated with cancer and its therapy, support preventive care, and guide ongoing surveillance across the life course [2, 3].

Survivorship care plans (SCPs) were developed to support this transition by summarizing a survivor’s treatment exposures and providing individualized recommendations for long-term follow-up. In principle, SCPs can improve survivor and provider knowledge, clarify care responsibilities, and support communication between oncology and primary care. However, implementation has remained challenging. Many pediatric cancer centers create treatment summaries or SCPs, but overall survivorship care delivery remains inconsistent, and resource constraints, limited staffing, and time-intensive preparation continue to limit broad and reliable use [4]. Evidence that SCPs improve patient outcomes or care delivery remains limited, with randomized trials and systematic reviews demonstrating little consistent benefit despite broad endorsement of their intended value [5–7].

Current SCP workflows often require clinicians to manually extract treatment details from electronic health records, legacy documents, and prior oncology notes to assemble a treatment summary and surveillance plan. This process can be labor-intensive, vulnerable to omissions, and difficult to sustain at scale [8–10]. Even when SCPs are created, they may not be easily accessible to survivors, families, or primary care providers. Because SCPs are intended to translate treatment history into coordinated follow-up care, their limitations extend beyond document creation. Prior studies have described specific limitations of SCPs, including gaps in communication between oncology and primary care, uncertainty about follow-up responsibilities, and unmet information needs among survivors and families [11–13].

These challenges suggest that SCP underuse reflects broader misalignments among survivorship workflows, information needs, clinical roles, and communication systems, rather than a lack of written information alone. Advances in health information technology, including artificial intelligence (AI)-assisted tools, may help address some of these structural barriers, but only if they are designed around the needs and workflows of the stakeholders expected to use them. Understanding how survivors, families, primary care providers, and oncology clinicians experience current SCPs is therefore necessary before developing new digital or AI-supported approaches.

In this qualitative study, we conducted semi-structured interviews with childhood cancer survivors and family members, primary care providers, and oncology clinicians to characterize how SCPs are created, shared, and used in pediatric oncology. We sought to identify barriers to current SCP use and stakeholder priorities for improving SCP workflows, content, and future digital or AI-supported tools. By examining stakeholder experiences with current SCP practices, this study aims to identify concrete targets for improving SCP usability, communication, workflow integration, and role-specific design.

## 2 | Methods

### 2.1 | Study Design and Participants

We conducted a qualitative study using semi-structured interviews with three stakeholder groups involved in pediatric cancer survivorship care: childhood cancer survivors and family members, primary care providers (PCPs), and pediatric oncology clinicians. The study was designed to characterize experiences with SCPs, identify barriers to SCP use, and elicit stakeholder priorities for future SCP workflows and tools. We used an inductive thematic analytic approach, with later interpretation informed by a sociotechnical work-system perspective [14, 15].

Participants were recruited from a single academic medical center, Monroe Carell Jr. Children’s Hospital at Vanderbilt, and affiliated clinical networks. We used purposive sampling to capture perspectives across stakeholder groups and professional roles, with an initial target of 5–10 participants per group. Survivor and family recruitment was intentionally limited to childhood leukemia and lymphoma survivors to provide a focused initial evaluation within a defined clinical population with common treatment exposures. Survivor and family participants were eligible if the survivor was diagnosed between ages 1 and 21 years, had completed curative-intent therapy between 2017 and 2024, and was either within 2 months of the end of therapy or engaged in long-term survivorship follow-up. Survivor and family participants were approached during routine oncology or survivorship clinic visits. Because most survivors approached were younger than 18 years, and caregivers utilized the SCPs, caregivers commonly served as the interview participants. Although adult survivors were eligible for participation and some expressed initial interest, only one ultimately completed an interview. PCPs were identified through institutional primary care networks and a regional pediatric practice consortium. Pediatric oncology clinicians were recruited from the pediatric hematology-oncology division. Recruitment continued until the study team determined that the sample provided sufficient depth across stakeholder groups to address the study objectives and that additional interviews were yielding limited new concepts. Participants received a $75 gift card for their time.

### 2.2 | Data Collection

The lead investigator (M.T.), a pediatric hematology-oncology fellow, conducted all interviews between February 2025 and September 2025 using secure, Health Insurance Portability and Accountability Act (HIPAA)-compliant video conferencing. Interviews were conducted individually and lasted approximately 30–45 min. Three semi-structured interview guides were developed, one for each stakeholder group, with input from experienced qualitative researchers and review of prior literature on survivorship care planning, communication, and care coordination. The guides explored experiences with SCP receipt or creation, perceived usefulness, barriers to use, communication across care settings, and suggestions for improvement. Interview guides are provided in Supporting Materials S1.

Interview guides were shared with participants in advance to allow reflection before the interview. Interviews were audio-recorded through Microsoft Teams, and an initial transcript was generated using the platform’s automated transcription function. The interviewer reviewed each transcript against the recording and edited the transcript for accuracy, including correction of transcription errors and removal of identifying information before analysis. Because the interviewer was a pediatric oncology clinician and researcher, she used reflexive strategies during data collection and analysis to consider how her clinical role could influence participant responses, questioning, and interpretation. These included use of a semi-structured interview guide, neutral follow-up prompts, transcript review, team-based codebook refinement, and discussion of emerging interpretations with investigators from clinical informatics, human factors, and pediatric oncology.

### 2.3 | Data Analysis

Transcripts were analyzed using inductive thematic analysis with constant comparison across stakeholder groups [15]. The lead investigator first reviewed a subset of transcripts and performed open coding to develop an initial codebook. The codebook was refined through discussion with the study team and updated iteratively as new concepts emerged. Coding was managed in Dedoose v9.0 (SocioCultural Research Consultants, Los Angeles, CA).

Coding was conducted by three investigators with complementary expertise: M.T., the lead investigator; L.L.N., a qualitative research and human factors expert; and E.F., a pediatrics resident with prior qualitative research experience. More than 20% of transcripts were independently double-coded by two co-analysts to assess consistency and refine code definitions. Coding discrepancies were resolved through discussion and consensus, with codebook revisions documented in Dedoose as an electronic audit trail. The study team met regularly to review emerging patterns, compare perspectives across survivor/family, PCP, and oncology clinician participants, and develop higher order themes. Representative quotations were selected to illustrate each theme and were lightly edited for readability without changing meaning.

After initial theme development, the team interpreted findings using a sociotechnical work-system lens to examine how SCP use was shaped by interactions among people, tasks, tools, clinical roles, and organizational workflows [14, 16]. This interpretive step was used to organize the implications of the findings, rather than to impose predetermined codes during initial analysis.

### 2.4 | Ethical Considerations

This study was approved by the Vanderbilt University Medical Center Institutional Review Board (IRB 241788). Informed consent was obtained from all participants. For minors, parental consent and child assent were obtained consistent with institutional and federal requirements. Interview recordings and transcripts were stored securely on encrypted institutional servers, and all transcripts were de-identified before analysis. This study was not designed as a clinical trial and therefore does not have a clinical trial registration number.

## 3 | Results

We interviewed 21 participants, including seven survivor or family participants, nine primary care providers, and five pediatric oncology clinicians. The survivor and family group included one young adult survivor and six family caregivers, all connected to acute lymphoblastic leukemia diagnoses. Primary care participants included five academic physicians, one academic nurse practitioner, and three community-based physicians, and oncology participants included three nurse practitioners and two attending oncologists. Participant characteristics are summarized in Table 1.

Analysis identified four higher order themes describing how stakeholders experienced current SCP practices and elements they viewed as necessary for improvement: (i) survivorship care remains oncology-centered, reflecting relational continuity developed during treatment and uncertainty about shared-care goals with primary care; (ii) SCPs are valued but poorly integrated into clinical workflows; (iii) SCP content reflects standardized documentation more than tailored user needs; and (iv) stakeholders viewed digital and AI-assisted tools as potentially helpful for reducing manual work and improving access, but emphasized accuracy, transparency, and clinician oversight. Themes and representative quotations are summarized in Table 2.

### 3.1 | Theme 1: Survivorship Care Remains Oncology-Centered Despite Shared-Care Goals

Across stakeholder groups, participants described survivorship care as strongly anchored in the oncology team. Families often continued to view oncology clinicians as the primary source of medical guidance after treatment completion, reflecting trust developed during cancer therapy. PCPs recognized this pattern and described families as more likely to contact oncology for survivor-specific concerns because “their oncologist knows them best” and families “go straight back to oncology for most things” (PCP-3).

PCPs varied in their comfort with survivorship follow-up. Some described an active role in routine preventive care, immunizations, and helping families remain connected to recommended oncology follow-up. One PCP summarized this role as making sure survivors were “meeting their cadence of follow-up” and helping reconnect families with oncology if they had been lost to care (PCP-2). However, PCPs also described uncertainty about which aspects of survivorship care they were expected to manage independently. Oncology clinicians similarly perceived that PCPs often preferred specialist guidance for cancer-related follow-up. One oncology provider noted that PCPs “don’t really feel as comfortable managing those aspects” and “prefer to defer to us because we are the experts” (Oncology Provider-1).

For families, this oncology-centered pattern reflected relational continuity built during treatment rather than a lack of PCP willingness to participate in survivorship care. For PCPs, it reflected structural ambiguity about role boundaries that existing SCP processes did not resolve. As a result, survivorship care responsibilities were not always clearly distributed across oncology, primary care, and families.

### 3.2 | Theme 2: SCPs Are Valued But Poorly Integrated Into Clinical Workflows

Participants generally endorsed the purpose of SCPs, but described limited use in day-to-day care. Families appreciated having a written summary of cancer treatment and follow-up recommendations, but many did not consistently refer back to the SCP or know where to find it when questions arose. Oncology clinicians also saw potential value in SCPs, particularly when families needed details such as treatment dates, exposures, or follow-up schedules. One provider explained that if a family “lost their copy or can’t find it,” it would be helpful for the SCP to be accessible in the chart for future reference (Oncology Provider-1).

Despite this perceived value, SCPs were inconsistently shared and difficult to access across care settings. PCPs commonly reported not receiving SCPs. One PCP stated, “I don’t think I’ve really received anything like this directly to me” (PCP-8). Oncology clinicians described sending SCPs by fax or providing printed copies to families, but were unsure whether PCP offices received or reviewed them. These workflows left families responsible for storing and transporting information that was intended to support provider-to-provider communication.

Clinicians also described SCP creation as manual and time-consuming. Oncology providers reported searching through multiple areas of the medical record to locate treatment dates, protocols, chemotherapy exposures, procedures, and surveillance recommendations. One clinician described having to “manually go in and pull in a bunch of information that’s difficult to find” (Oncology Provider-3). Another noted that when using a general template, “none of [the data] gets pulled in,” leaving clinicians to find the information themselves or leave sections blank (Oncology Provider-4). PCPs experienced the downstream effects of these gaps when SCPs were missing, delayed, or incomplete. One PCP described spending “20–30 min just digging through notes” before a visit to reconstruct the survivor’s history (PCP-1).

Together, these findings suggest that SCPs were viewed as useful in principle, but were not reliably embedded into the workflows needed to make them useful in practice. Across groups, participants described a need for SCP processes that were easier to maintain, share, and retrieve. Oncology clinicians and PCPs additionally emphasized the need to reduce manual data-gathering burden during SCP creation.

### 3.3 | Theme 3: SCP Content Reflects Standardized Documentation More Than Tailored User Needs

A consistent finding across stakeholder groups was that current SCPs are structured around clinical documentation conventions rather than around the varying needs of the people expected to use them. Families, PCPs, and oncology clinicians all identified content gaps and format mismatches, but the nature of those mismatches differed by user.

Families often characterized SCP materials as dense, generic, or difficult to interpret. One caregiver described receiving “a big, bifold cardboard piece” that “felt kind of generic” (Parent-1). Another described being given a binder with so much information that they “probably wouldn’t know what [the medicines] were anyway” (Parent-3). For some families, the volume and tone of information were not only difficult to use but emotionally overwhelming. One caregiver described the survivorship binder as “so overwhelming” that reviewing it could feel distressing (Parent-4).

Clinicians recognized that current SCPs often seemed designed more for providers than for families. One oncology provider described the existing format as “very provider-centric” and suggested that if the SCP is being handed to families, “maybe it should look different for them” (Oncology Provider-4). PCPs similarly wanted concise and actionable information that could be used efficiently in primary care. Rather than lengthy treatment summaries alone, they wanted clear surveillance recommendations, responsibility delineation, and guidance on when to refer back to oncology.

Participants also identified gaps in SCP content beyond biomedical surveillance. Families and PCPs described ongoing needs related to mental health, school functioning, fatigue, neurocognitive concerns, fertility, and other psychosocial or functional effects of cancer treatment. One caregiver described feeling in “a little bit of a black hole” when trying to find mental health support for cancer-related post-traumatic stress disorder (PTSD) and depression (Parent-4). PCPs noted that many survivorship-related concerns they encountered involved school problems, fatigue, cognitive issues, anxiety, or depression, but they were not always sure whether these concerns were treatment-related or how they should be addressed.

These findings suggest that improving SCP content requires more than adding information. Participants described a need for role-specific organization: plain-language guidance for survivors and families, concise action items for PCPs, and sufficiently detailed treatment and guideline information for oncology teams.

### 3.4 | Theme 4: Stakeholders Viewed Technology-Enabled SCPs as Potentially Helpful But Emphasized Transparency and Clinician Oversight

Stakeholders identified potential benefits of digital tools and AI-assisted approaches for improving SCP creation and use. Clinicians saw potential for technology to reduce manual data entry, pull treatment information from the electronic health record (EHR), and help keep recommendations aligned with current survivorship guidelines. PCPs similarly valued the possibility of plans that contained “the most recent and updated information” to guide care (PCP-9).

However, participants emphasized that accuracy, transparency, and clinician oversight would be essential. Families and clinicians raised concerns about whether AI-generated information could be trusted. One caregiver asked “how accurate” an AI-developed plan would be and whether it could be trusted (Parent-4). A PCP suggested that an AI-supported SCP should include a clear indication of when it was drafted and when it was reviewed by an oncologist, such as a timestamp showing “drafted by AI on this date, reviewed by oncologist on this date” (PCP-9). This visible review process was viewed as important for trust, accountability, and safe use.

Participants also emphasized that technology should not replace the relational aspects of survivorship care. One PCP noted that survivorship includes an “emotional psychological journey” that may not be fully captured by AI (PCP-9). At the same time, digital access was viewed as valuable, particularly for making SCPs easier to store and reference over time. The young adult survivor participant described wanting a plan that could be kept “in the files on my phone,” because it would be easier to reference and “everything is digital now” (Patient-1).

Overall, stakeholders viewed technology as a tool to support, rather than replace, survivorship care. They saw the greatest value in tools that could reduce manual work, improve access to updated information, and make SCPs more usable across settings while preserving clinician review and human communication.

## 4 | Discussion

In this qualitative study of survivor and family participants, primary care providers, and pediatric oncology clinicians, we found that SCPs were valued in principle, but were not consistently integrated into the workflows needed to make them useful in practice. Four findings were central: survivorship care remained strongly oncology-centered despite shared-care goals; SCPs were difficult to create, share, retrieve, and update; SCP content often reflected documentation needs more than user needs; and participants identified potential benefits of digital and AI-assisted tools but emphasized transparency and clinician oversight. Together, these findings suggest that SCP underuse is not simply a problem of awareness or dissemination. Rather, current SCPs are expected to coordinate complex, lifelong survivorship care while functioning as static documents within fragmented systems of communication, responsibility, and information exchange.

Prior studies have described variable SCP implementation, limited PCP receipt of SCPs, unmet information needs among survivors and families, and uncertainty about the role of primary care in childhood cancer survivorship [4, 11, 12]. Evidence that SCPs improve outcomes or care delivery has also remained limited despite broad support for their intended purpose [5–7]. Our findings extend this literature by examining how these barriers operate dynamically across survivor and family participants, PCPs, and oncology clinicians within a single survivorship system. Participants did not describe SCPs as unimportant. Instead, they described SCPs as poorly aligned with the real work of survivorship care, including locating treatment information, clarifying responsibilities, communicating across settings, and helping families understand and act on long-term recommendations.

Interpreting these findings through a sociotechnical work-system lens helps explain why SCPs may remain underused even when stakeholders value them. The Systems Engineering Initiative for Patient Safety (SEIPS) 3.0 Model provides a human factors framework for examining how care processes unfold across the patient journey, emphasizing that outcomes emerge from interactions among people, tasks, tools, technologies, organizational structures, and environments and across multiple work systems [14]. From this perspective, an SCP is not merely a document. It is a tool intended to support coordination among survivors, families, oncology teams, PCPs, medical records, guidelines, and clinical workflows. In our study, the SCP often did not function as an effective work-system tool. Oncology clinicians spent substantial time manually assembling information, PCPs lacked reliable access to plans or clarity about their role, and families were often left to store, interpret, and transmit information across settings. These findings suggest that improving SCPs requires redesigning not only the document but also the surrounding processes that determine who creates it, who receives it, when it is updated, and how it is used.

One important implication is the need to clarify survivorship roles within oncology and primary care while preserving the relational continuity families value. Families’ reliance on oncology teams reflected trust and continuity built during treatment and the perception that oncology clinicians best understood the survivor’s history. This oncology-centered pattern should not be interpreted as evidence that PCPs are unwilling to care for childhood cancer survivors. Rather, PCPs described uncertainty about what they were responsible for managing, limited access to survivor-specific information, and uncertainty about when they should defer to or re-engage oncology. This ambiguity can limit shared care and may increase reliance on families as intermediaries between clinicians. Prior work has similarly shown that PCPs are willing to participate in survivorship care, but need clear guidance, treatment summaries, and delineation of responsibilities [11, 12]. SCPs could help fill this gap, but only if they move beyond listing recommendations to explicitly assign responsibility for specific surveillance, preventive care, immunizations, referrals, and symptom evaluation.

A second implication is that SCPs should be redesigned around the needs of distinct users rather than as a single static document. Participants described current SCPs as dense, generic, difficult to navigate, and at times emotionally overwhelming. PCPs wanted concise, actionable recommendations that could be used during routine visits, while families wanted information that was understandable, accessible, and relevant to their lived experience after treatment. Oncology clinicians required sufficiently detailed treatment and guideline information to support accurate risk-based follow-up. A more useful approach may therefore involve layered or role-specific SCP outputs: a plain-language version for survivors and families, a concise clinical action plan for PCPs that includes explicit responsibility assignments, and a more detailed treatment and guideline record for oncology teams. This approach may also better address psychosocial and developmental concerns, including school functioning, fatigue, neurocognitive effects, fertility, and mental health needs, which participants viewed as important but inconsistently represented in current SCPs.

Digital tools may help support this user-centered redesign by improving access, reducing manual data gathering, and enabling SCP content to be updated and presented differently for distinct users. Prior work suggests that health information technology can reduce the time required to generate SCPs, but that existing systems have not fully leveraged electronic sharing, communication, and care-coordination functions across the care team [17]. Generative AI and other AI-assisted approaches may offer one potential mechanism for summarizing treatment information and generating role-specific outputs. Participants viewed these approaches as potentially useful for reducing manual work and improving access to updated information, while emphasizing accuracy, transparency, visible clinician review, and preservation of human communication. These findings should be interpreted as preliminary design requirements and priorities for future evaluation rather than evidence of broad stakeholder acceptability. Future work should assess whether digital and AI-assisted approaches can produce accurate, usable, equitable, and appropriately tailored outputs while supporting clinician judgment and relational continuity.

These findings have practical implications for future SCP design. SCPs should be embedded within clinical workflows through reliable EHR access, direct communication to PCPs, and mechanisms for updating plans across survivorship visits. Rather than functioning as isolated documents, SCPs should provide role-specific recommendations and explicit responsibility assignments that clarify what oncology teams, primary care providers, and families are expected to do. Content should be organized for usability, with plain-language explanations, prioritized action items, and guidance for psychosocial and functional concerns. Digital and AI-assisted systems may help operationalize these goals, but their value should be assessed according to whether they improve access, usability, role clarity, communication, and continuity of care.

## 5 | Limitations and Future Directions

This study has several limitations. It was conducted at a single academic pediatric oncology center with an established survivorship program, which may limit transferability to institutions with different survivorship models or fewer resources. The survivor and family sample was intentionally limited to childhood leukemia and lymphoma survivors, and all enrolled survivor or family participants were connected to acute lymphoblastic leukemia diagnoses. Most of the survivor population who consented were caregivers of those younger than 18 years. The findings therefore may not reflect the experiences of survivors with other diagnoses or treatment exposures, particularly survivors of brain tumors or hematopoietic stem cell transplantation, who may have distinct and often highly complex neurocognitive, functional, psychosocial, and multispecialty needs. The survivor and family group also primarily reflected caregiver perspectives, with only one young adult survivor participant, limiting conclusions about survivors’ direct experiences as they assume greater responsibility for their own care. Non-English-speaking families were not represented.

Because interviews relied on participant self-report, responses may have been affected by recall bias or by participants’ prior experiences with this institution. The interviewer’s role as a pediatric oncology clinician may have shaped participant responses and interpretation of the data; we attempted to mitigate this through use of a semi-structured guide, transcript review, team-based coding, and multidisciplinary discussion of emerging themes. In addition, perspectives on AI-assisted tools may evolve as these technologies become more familiar in clinical care, and the limited number of participants commenting directly on AI restricts the strength of conclusions that can be drawn about its acceptability.

Despite these limitations, the study provides a stakeholder-informed foundation for redesigning SCPs as part of a broader survivorship work system. Future work should include larger and more diverse survivor populations, including survivors of other cancer diagnoses, adult survivors of childhood cancer, non-English-speaking families, and community-based practices. Future studies should also evaluate whether redesigned SCP tools improve access, comprehension, role clarity, communication, and follow-up completion. Our next step is to use these findings to inform development of a digital SCP prototype that automates treatment data compilation where feasible, integrates current guideline logic, and uses AI-assisted functions to generate role-specific outputs for survivors, families, PCPs, and oncology clinicians. Evaluation should include not only technical accuracy but also usability, trust, cognitive burden, and the extent to which the tool supports shared survivorship care.

## 6 | Conclusion

SCPs remain important tools for pediatric cancer survivorship, but their current form does not consistently support the work they are intended to accomplish. Stakeholders valued the concept of SCPs but described persistent barriers related to access, workflow integration, role clarity, usability, and content relevance. Viewing SCPs as tools within a survivorship work system highlights why improving the document alone is unlikely to be sufficient. Future SCP design should prioritize role-specific content, explicit responsibility assignments, reliable communication across settings, and integration into clinical workflows. Digital and AI-assisted approaches may help support these goals, but should preserve clinician oversight and the relational continuity that survivors and families depend on throughout the cancer journey.

## Supplementary Material

Supplemental Materials

Additional supporting information can be found online in the Supporting Information section.

**Supporting File**: pbc70677-sup-0001-SuppMat.docx.

## Figures and Tables

**TABLE 1 |:** Participant characteristics.

Stakeholder group	*n*	Role or relationship	Diagnosis	Clinical specialty
Survivors/Family members	7	1 patient, 6 parents	Acute lymphoblastic leukemia (7)	
Primary care providers (PCPs)	9	3 community practice, 6 academic		General pediatrics (7) Medicine-pediatrics (1) Internal medicine (1)
Oncology clinicians	5	3 nurse practitioners, 2 attending oncologists		Survivorship (2) Pediatric leukemia/lymphoma (3)
Total	21			

**TABLE 2 |:** Higher order themes and representative quotations from stakeholder interviews.

Higher order theme	Key finding	Representative quotations
1. Survivorship care remains oncology-centered despite shared-care goals	Families continued to rely heavily on oncology teams because of relational trust built during treatment, while PCPs and oncology clinicians described unclear role boundaries in survivorship follow-up	“Our primary care has been with the oncologist really more so than our pediatrician, other than the regular sick visits.” Family Caregiver-4 “It’s just sometimes hard to know what I’m supposed to be in charge of versus a subspecialist.” PCP-1 “I don’t feel like we communicate with primary care providers well across the board. I think we sort of take over their care in such a way that we are their PCP for the whole duration.” Oncology Provider-3
2. SCPs are valued but poorly integrated into clinical workflows	SCPs were viewed as useful in principle, but participants described inconsistent access, unreliable sharing across settings, and manual data-gathering burden during creation and use	“I don’t think I’ve really received anything like this [SCP] directly to me.” PCP-8 “They don’t pull in a lot of the data at all… you’re having to sort of manually go in and pull in a bunch of information that’s difficult to find and to locate.” Oncology Provider-3 “I really think it should be both. I appreciate the paper document and being handed it at the survivorship appointment. I actually think it should be given at every survivorship appointment with updates.” Family Caregiver-5
3. SCP content reflects standardized documentation more than tailored user needs	Current SCPs were often perceived as dense, generic, provider-centric, or incomplete for the practical, psychosocial, and developmental needs of families and PCPs	“They’ve loaded us up with so much information… I just do what I’m told… I probably wouldn’t know what [the medicines] were anyway.” Family Caregiver-3 “This [current SCP format] looks very provider-centric… something that we are using. If it’s something you’re handing to the family, maybe it should look different for them.” Oncology Provider-4 “Most commonly, I feel like it’s school problems, fatigue or cognitive issues… being unsure if it’s related to treatment, or if it’s expected.” PCP-2
4. Stakeholders viewed technology-enabled SCPs as potentially helpful, but emphasized transparency and clinician oversight	Participants saw potential for digital and AI-assisted SCPs to improve updating, access, and efficiency, but emphasized accuracy, trust, and preservation of human review	“The COG… come out with new updates… when the most recent update came out… there were some things that we needed to change on our care plans.” Oncology Provider-5 “Maybe like a timestamp, like drafted by AI on this day, reviewed by primary oncologist on this day… some indication of if and when it was reviewed by a human.” PCP-9 “I would probably keep it in the files on my phone, just cause it’s easier for me to reference that and everything is digital now… I think it would be really great.” Young Adult Survivor-1

*Note:* Quotes are lightly edited for clarity and de-identified by stakeholder role.

Abbreviations: COG, Children’s Oncology Group; PCP, primary care provider; SCP, survivorship care plan.

## Abbreviations:

AI: artificial intelligence
EHR: electronic health record
HIPAA: Health Insurance Portability and Accountability Act
IRB: Institutional Review Board
PCP: primary care provider
PTSD: post-traumatic stress disorder
SCP: survivorship care plan
SEIPS: Systems Engineering Initiative for Patient Safety
