# Supplementary material for: Beyond the Document: A Single-Center Qualitative Study of Survivorship Care Plan Barriers and Opportunities Across Pediatric Oncology Stakeholders
Source: Pediatr Blood Cancer. Author manuscript; Available in PMC 2026 Sep 22. (PMC13591526; doi:10.1002/1545-5017.70677)
Supplement: Supplementary file 1 [file NIHMS2209898-supplement-Supplemental_Materials.docx]

**Supplement 1: Interview Guides**

Semi-Structured Interview Script – Patients/Families:

**Hello, my name is [*first name, last name*]. We are working with your providers at Vanderbilt University Medical Center. Thank you for agreeing to participate in this study. We will ask questions about cancer survivorship. This interview will take about 15-30 minutes. From time to time, we will ask you follow-up questions about your/your child’s health or if we need your help to fill in missing or unclear information. You and/or your child are free to skip any questions that you do not wish to answer. At the end of the interview, you will have the option to be contacted for future involvement in other parts of the study.**

Survivorship Care Plans are documents developed by an oncology care team to facilitate communication among oncologists, patients, and primary care doctors after cancer treatment and to assist cancer survivors with healthcare decisions. These contain on treatment exposures and toxicities and recommended monitoring for adverse health outcomes. I am going to ask you some questions about you/your child’s experience with survivorship care plans.

1. What information have you received regarding your/your child’s cancer and needs after completing treatment?
   a. How adequate is this information?
   b. What additional information do you need?
2. What are your greatest concerns about providing care for yourself/your child after completion of cancer therapy?
3. Do you feel that a survivorship care plan is/would be useful? How so?
4. What suggestions do you have for improvement of a survivorship care plan?
5. What is your preferred format for a survivorship care plan?
6. Would you be willing to be contacted for additional interviews or study questions related to development of new survivorship care plan prototypes?

Semi-Structured Interview Script – PCPs:

**Hello, my name is [*first name, last name*]. We are working with providers at Vanderbilt University Medical Center. I am calling to speak to [title, first name, last name] about our study you indicated interest in. Thank you for agreeing to participate in this study. We will ask questions about cancer survivorship. This interview will take about 15-30 minutes. You are free to skip any questions that you do not wish to answer. At the end of the interview, you will have the option to be contacted for future involvement in other parts of the study.**

Survivorship Care Plans are documents developed by an oncology care team to facilitate communication among oncologists, patients, and primary care doctors after cancer treatment and to assist cancer survivors with healthcare decisions. These contain on treatment exposures and toxicities and recommended monitoring for adverse health outcomes. I am going to ask you some questions about your experience with survivorship care plans for your patients.

1. What is your current practice in taking care of survivors of cancer?
2. What do you perceive your role is in the long-term management of cancer survivors?
3. What information have you received regarding your patient’s cancer and needs after completing treatment?
   a. How adequate is this information?
   b. What additional information do you need from oncologists?
4. Do you feel that a survivorship care plan is/would be useful? How so?
5. What suggestions do you have for improvement of a survivorship care plan?
6. What is your preferred format for a survivorship care plan?
7. Would you be willing to be contacted for additional interviews or study questions related to development of new survivorship care plan prototypes?

Semi-Structured Interview Script – Oncologists/Oncology Nurses:

**Hello, my name is [*first name, last name*]. We are working with providers at Vanderbilt University Medical Center. I am calling to speak to [title, first name, last name] about our study you indicated interest in. Thank you for agreeing to participate in this study. We will ask questions about cancer survivorship. This interview will take about 15-30 minutes. You are free to skip any questions that you do not wish to answer. At the end of the interview, you will have the option to be contacted for future involvement in other parts of the study.**

Survivorship Care Plans are documents developed by an oncology care team to facilitate communication among oncologists, patients, and primary care doctors after cancer treatment and to assist cancer survivors with healthcare decisions. These contain on treatment exposures and toxicities and recommended monitoring for adverse health outcomes. I am going to ask you some questions about your experience with survivorship care plans for your patients.

1. What is your current practice in taking care of survivors of cancer?
2. What do you perceive your role is in the long-term management of cancer survivors?
3. What information do you need from oncologists to take care of your patients who have survived childhood cancer and completed treatment?
4. Do you feel that a survivorship care plan is/would be useful? How so?
5. What suggestions do you have for improvement of a survivorship care plan?
6. What is your preferred format for a survivorship care plan?
7. Would you be willing to be contacted for additional interviews or study questions related to development of new survivorship care plan prototypes?

**Supplement 2: COnsolidated criteria for REporting Qualitative studies (COREQ): 32-item checklist**

Developed from Tong A, *et al.*

The checklist reports how the study addressed COREQ items. Items not performed or not systematically collected are noted as such.

| **Item number** | **Guide questions/description** | **Reported on page number** |
| --- | --- | --- |
| **Domain 1: research team and reflexivity** | | |
| **Personal characteristics** | | |
| 1. Interviewer/facilitator | Which author/s conducted the interview or focus group? | p. 7 |
| 2. Credentials | What were the researcher’s credentials (e.g. PhD, MD)? | p. 1, 7 |
| 3. Occupation | What was their occupation at the time of the study? | p. 7 |
| 4. Gender | Was the researcher male or female? | p. 7 (“she”) |
| 5. Experience and training | What experience or training did the researcher have? | p. 7 |
| **Relationship with participants** | | |
| 6. Relationship established | Was a relationship established prior to study commencement? | Not systematically recorded; partially addressed p. 7 |
| 7. Participant knowledge of the interviewer | What did the participants know about the researcher (e.g. personal goals, reasons for doing the research)? | p. 7 |
| 8. Interviewer characteristics | What characteristics were reported about the interviewer/facilitator (e.g. bias, assumptions, reasons and interests in the research topic)? | p. 7, p. 19 |
| **Domain 2: study design** | | |
| **Theoretical framework** | | |
| 9. Methodological orientation and Theory | What methodological orientation was stated to underpin the study (e.g. grounded theory, discourse analysis, ethnography, phenomenology, content analysis)? | p. 6, p. 8 |
| **Participant selection** | | |
| 10. Sampling | How were participants selected (e.g. purposive, convenience, consecutive, snowball)? | p. 6 |
| 11. Method of approach | How were participants approached (e.g. face to face, telephone, mail, e-mail)? | p. 6 |
| 12. Sample size | How many participants were in the study? | p. 9; Table 1 p. 21 |
| 13. Non-participation | How many people refused to participate or dropped out? Reasons? | Not systematically collected |
| **Setting** | | |
| 14. Setting of data collection | Where was the data collected (e.g. home, clinic, workplace)? | pp. 6-7 |
| 15. Presence of non-participants | Was anyone else present besides the participants and researchers? | Not systematically recorded |
| 16. Description of sample | What are the important characteristics of the sample (e.g. demographic data, date)? | p. 9; Table 1 p. 21 |
| **Data collection** | | |
| 17. Interview guide | Were questions, prompts, guides provided by the authors? Was it pilot tested? | p. 7; Supplemental Material 1; not pilot tested |
| 18. Repeat interviews | Were repeat interviews carried out? If yes, how many? | No repeat interviews |
| 19. Audio-/visual-recording | Did the research use audio or visual recording to collect the data? | p. 7 |
| 20. Field notes | Were field notes made during and/or after the interview or focus group? | Not systematically collected |
| 21. Duration | What was the duration of the interviews or focus group? | p. 7 |
| 22. Data saturation | Was data saturation discussed? | p. 6 |
| 23. Transcripts returned | Were transcripts returned to participants for comment and/or correction? | Transcripts not returned to participants |
| **Domain 3: analysis and findings** | | |
| **Data analysis** | | |
| 24. Number of data coders | How many data coders coded the data? | p. 8 |
| 25. Description of the coding tree | Did authors provide a description of the coding tree? | p. 8 |
| 26. Derivation of themes | Were themes identified in advance or derived from the data? | p. 8 |
| 27. Software | What software, if applicable, was used to manage the data? | p. 8 |
| 28. Participant checking | Did participants provide feedback on the findings? | Participant checking not performed |
| **Reporting** | | |
| 29. Quotations presented | Were participant quotations presented to illustrate the themes/findings? Was each quotation identified (e.g. participant number)? | Results pp. 10-15; Table 2 pp. 22-25 |
| 30. Data and findings consistent | Was there consistency between the data presented and the findings? | Results pp. 9-15; Table 2 pp. 22-25 |
| 31. Clarity of major themes | Were major themes clearly presented in the findings? | Results pp. 9-15; Table 2 pp. 22-25 |
| 32. Clarity of minor themes | Is there a description of diverse cases or discussion of minor themes? | Results pp. 10-15 |

MD, Doctor of Medicine; n/a, not applicable
